# Context-Aware Human Activity Recognition in Industrial Processes

**DOI:** 10.3390/s22010134

**Published:** 2021-12-25

**Authors:** Friedrich Niemann, Stefan Lüdtke, Christian Bartelt, Michael ten Hompel

**Affiliations:** 1 Chair of Materials Handling and Warehousing, TU Dortmund University, Joseph-von-Fraunhofer-Str. 2-4, 44227 Dortmund, Germany; michael.tenhompel@tu-dortmund.de; 2 Institute for Enterprise Systems, University of Mannheim, L15 1, 68131 Mannheim, Germany; luedtke@es.uni-mannheim.de (S.L.); bartelt@es.uni-mannheim.de (C.B.)

**Keywords:** context awareness, human activity recognition, context model, motion capture, warehousing, logistics, industrial processes

## Abstract

The automatic, sensor-based assessment of human activities is highly relevant for production and logistics, to optimise the economics and ergonomics of these processes. One challenge for accurate activity recognition in these domains is the *context-dependence* of activities: Similar movements can correspond to different activities, depending on, e.g., the object handled or the location of the subject. In this paper, we propose to explicitly make use of such context information in an activity recognition model. Our first contribution is a publicly available, semantically annotated motion capturing dataset of subjects performing order picking and packaging activities, where context information is recorded explicitly. The second contribution is an activity recognition model that integrates movement data and context information. We empirically show that by using context information, activity recognition performance increases substantially. Additionally, we analyse which of the pieces of context information is most relevant for activity recognition. The insights provided by this paper can help others to design appropriate sensor set-ups in real warehouses for time management.

## 1. Introduction

In production and logistics, time data of workers, resources and work objects are used to determine performance-based remuneration and to plan and control deadlines and schedules [1] (p. 573 ff.). The time data of workers, i.e., their movements, are mainly recorded using classical methods of time management. When using methods such as the REFA time study, trained REFA employees use a stopwatch to record workers movements in real-time [2]. This manual process is time-consuming and cost-intensive [3] (p. 84), [4] (p. 199). In addition, the time data can be subjectively influenced, depending on the experience of the REFA employees [4] (p. 196).

These methods of time management do not take into account the technical potential of wearable sensor technology or machine learning. Wearable or environmental sensors are already used for the fully automatic recording of movements [5]. Compared with manual methods, the use of sensors eliminates the subjective factors in data acquisition and significantly reduces both effort and cost. Analysing sensor data through machine learning, especially human activity recognition (HAR), provides the potential to determine time data uniformly and objectively [6,7].

State-of-the-art activity recognition methods in logistics are based on movement data, recorded by wearable sensors and optical marker-based motion capture (oMoCap) systems [8,9]. For a comprehensive analysis of logistics processes, a fine-grained, detailed description of activities is necessary. For example, instead of measuring performance during packaging as an overall process, more detailed information can be used to analyse sub-processes, such as reaching for packaging material, filling a box or adding a delivery note. From the analysed data, waste, unevenness in workloads and overburdening can be identified [10] (p. 67). However, when attempting to recognise increasingly fine-grained activities, recognition accuracy will typically decrease, as the recognition task becomes more and more challenging.

A general strategy to increasing classification performance is to collect additional data streams that allow discriminating between the fine-grained activities [11,12]. Specifically, in addition to the worker and their movements, process-relevant environmental information, the so-called *context*, can be recorded. For example, the movement data in [13] were extended by audio, location and phone status to improve the activity recognition performance. Dourish [14] considers activities as entities that take place within a context, but can also exist detached from it. Therefore, we distinguish between human movements, as the basis for HAR, and context, which is not necessary for HAR but can be used as additional data. Context includes information about location, time, identity, conditions and infrastructure from the subject and the physical environment [15].

**Figure 1:**
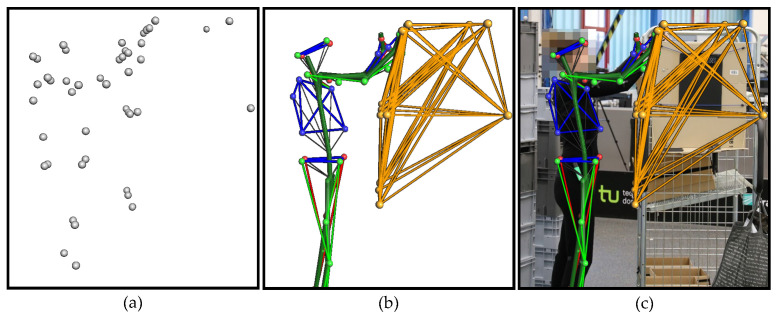
Data processing pipeline of the oMoCap system based on a person to goods order picking process: (**a**) Reconstruction of a point cloud consisting of markers. (**b**) Marker labelling based on patterns of markers from the subject and the picking cart. (**c**) Overlay of the oMoCap visualisation with the video recording.

This paper presents a context-aware model for HAR in logistics. The starting point for the development of the model is the first logistical dataset with context data for HAR—**CAARL** [16]: **C**ontext-**A**ware **A**ctivity **R**ecognition in **L**ogistics (Section 3). CAARL contains **human movement data** and **context data**. **The context includes the identities and locations of objects**. Like the subjects, 12 objects were equipped with reflective markers. Using an oMoCap system, the markers were captured and recorded. An exemplary overlay of the oMoCap visualisation with the video recording is shown in Figure 1. The markers make it possible to identify objects and sub-components of objects and to determine their locations. Since the location of the subject is also recorded, the distances between the subjects and all objects and sub-components can be determined. The context can be used as additional information to improve the performance of the HAR method based on motion data.

We use a model that allows such context information to be directly integrated into motion-based HAR. The model combines a pre-trained neural network that predicts high-level motion descriptions (attributes) with a classifier that predicts activity classes from these attributes and context information (Section 4). The approach is flexible in the sense that classifiers can be easily replaced and additional context information can be integrated without complete re-training. The empirical results show that the use of context information significantly improves the activity recognition performance in terms of F1 scores (Section 5). In addition, we analyse which context features are most relevant to increase the performance of activity recognition. The results of this analysis can be used to record only the most informative context information in a real warehouse. This greatly simplifies the entire time management process.

## 2. Related Work

### 2.1. Related Datasets

The *CAARL* dataset is based on the set-up of the freely available logistics dataset *LARa* [8,17]. Both datasets were recorded in the same laboratory environment and contain the same scenarios. They differ in the entities captured. While *LARa* only contains human movements, *CAARL* also includes objects.

The dataset *AndyData-lab-onePerson* [18,19] includes similar activities as the *LARa* dataset and can be assigned to logistics. Various postures of the legs, upper body and hands are labelled. With activity classes such as Reach, Pick, Place, Release, Carry etc., the posture of the hands is even more detailed than in *LARa*. Just like *LARa*, *AndyData-lab-onePerson* does not contain any context information. Thus, the only two freely accessible logistical datasets for the development of an intralogistic context-based approach are omitted.

Consequently, a new dataset had to be created. The *KIT Whole-Body Human Motion Database* [20,21] helped with orientation. The dataset cannot be assigned to only one domain. In addition to cooking activities, locomotion and healthcare activities were recorded using an oMoCap system. The recordings each include the movements of a subject and the marked objects with which the subject interacts. For the cooking activities, for example, a cucumber, whisk, pizza box, bowl, cup and a cooking spoon were recorded. Locomotion and grooming activities include, for example, a staircase, a seesaw and a sponge. *CAARL* is based on a similar approach, but involves a much larger laboratory environment with more complex processes. Furthermore, the datasets differ in their scenarios and the objects used.

The oMoCap system is not suitable for use in a real warehouse. However, due to its high accuracy, it serves as a reference and represents the ground truth with which more inaccurate sensors can be tested for their practicality. In the real warehouse, mobile sensors must be used, as was done, for example, in *Daily Log* [22,23], *RealWorld* [24] and *ExtraSensory Dataset* [13,25]. In these datasets, activities of daily living including locomotion activities were recorded using inertial measurement units and supplemented with location information from the GPS. Unlike the *ExtraSensory* approach, the location information from *Daily Log* was not used to improve activity accuracy. Instead, the linking of activities and location information helped to create a personal activity-position map and to optimise the daily routine concerning a healthier life. The *ExtraSensory Dataset* contains further context information in the form of audio files, which are also used for activity recognition.

From the approaches that have been implemented in the real world and not in the laboratory, the next step arises: The current approach needs to be extended to include mobile sensors.

### 2.2. Methods

Recently, deep neural networks have been very successful for sensor-based HAR [26,27]. The architecture described in Section 4 is a temporal convolutional network, as introduced in [28], i.e., it carries out a convolution and pooling operations along the time axis. Furthermore, our employed architecture is related to few-shot and transfer learning: We use a network that predicts domain-independent *movement attributes*, and predict activity classes based on the attributes. This allows one to recognise new, unseen activities, by making use of knowledge about the relationship between movement attributes and new activities. This concept was originally proposed for vision [29] and then adapted for HAR [30]. Specifically, attribute representations for HAR in a logistics context have been investigated in [31].

The use of *context* data to improve HAR has been considered in various forms before. For example, the authors of [32] used both ego-centric video data (to recognise used objects) and wearable sensor data for activity recognition in a warehouse scenario. The authors of [33] considered high-level process states as additional context information. However, the process states usually cannot be inferred directly from the sensor data. In our work, we make use of their proposed architecture, but use sensor-based context data instead of process states.

Furthermore, symbolic and hybrid HAR models [34,35,36] can also integrate context data. They model the causal structure of activities, e.g., via precondition-effect rules, and do not only estimate the currently performed activity, but maintain a distribution over *system states*, which can also include factors such as the locations or states of objects or subjects. In this way, integration of context data becomes straightforward, by extending the *observation model* (which relates system states to sensor data) appropriately. Such symbolic methods model the relationship between activities and context data explicitly, based on prior knowledge. Instead, our architecture learns the correspondence between activity classes and context directly from the available data.

## 3. The CAARL Dataset

Context information is already used to increase the recognition performance of HAR methods, e.g., in healthcare [37,38]. However, this idea does not apply to all domains. In logistics, context-aware activity recognition is unexplored, which is also reflected in the existing datasets. Logistic datasets, such as [17,18], only contain sensor data of human movements. Specifically, it has not been investigated systematically *which* context data is most relevant in (intra-)logistics to increase HAR performance. Here, a trade-off must be made between HAR performance (requiring rich, highly informative context features) and practical feasibility of recording (requiring low-cost data collection).

In order to determine which context data is relevant, we have recorded a rich dataset for Context-Aware Activity Recognition in Logistics (CAARL) that enables systematic investigation. CAARL contains human movement and object data. The information on the locations of objects in three-dimensional space can be categorised under what is being described as context. A marker-based oMoCap system was used to record both subjects and objects. In total, the CAARL dataset comprises 140 min of annotated recordings of two subjects and 12 objects. The entire dataset is freely available [16].

### 3.1. Laboratory Set-Up and Scenarios

**Figure 2:**
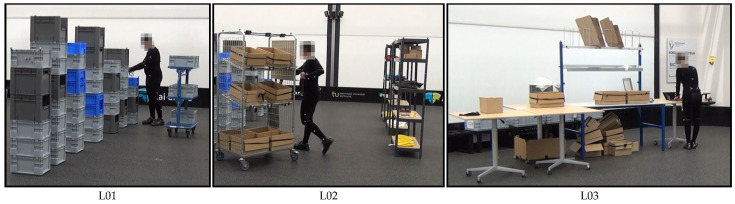
Extracts from the physical laboratory set-up of all three logistics scenarios.

Intralogistics processes were physically recreated in a laboratory set-up. Figure 2 shows examples of two different order picking scenarios and one packaging scenario.

Logistics scenario one (L01) was a simplified order picking system. Mainly, small items were picked from racks or from boxes stacked on top of each other. Then, they were placed on a small picking cart in small load carriers. Finally, the filled small load carriers were placed on a base. This scenario was not based on a real warehouse. However, its process steps were comparable to typical person-to-goods picking systems.

Logistics scenario two (L02) was based on a real-world order picking and consolidation system. The primary process remains item picking. Compared to the first scenario, the second scenario was extended with new process steps and the items used in them. The picked items were scanned using barcodes and then placed in boxes on a large picking cart. The subject had to confirm with a button on the put-to-light frame at the cart each time an item was placed in the box. When the box was filled for the first time, the subject had to mark the box with a stamp. This allows mistakes to be traced back to the worker. After all items were picked, the consolidation took place. The subject placed all boxes in the channels of the flow through rack.

In the third logistics scenario (L03), a physical simulation of the real-world packaging process was carried out. In this scenario, the items picked in the second scenario were carried from the flow rack to the packaging table, checked by means of a scale and repacked if necessary. The box was then packed ready for dispatch. This included filling the boxes with a layer of bubble wrap and one delivery note, and attaching a barcode to the outside of the box.

A detailed description of the three logistics scenarios can be found in [8] (pp. 6–12).

### 3.2. Human Movement Data—Subjects

One female subject aged 26 and one male subject aged 30 participated in the data collection. The specifications of the subjects are listed in Table 1. Both subjects were recorded for 70 min. The recordings are divided into two-minute sequences. Five recordings from the first scenario, 15 recordings from the second and 15 recordings from the third scenarios are available for each subject.

Each subject wore a suit, a headband and safety shoes, to which a total of 39 optical markers were attached (see Figure 2). The exact marker configuration is detailed in the recording protocol.

### 3.3. Context Data—Objects

**Figure 3:**
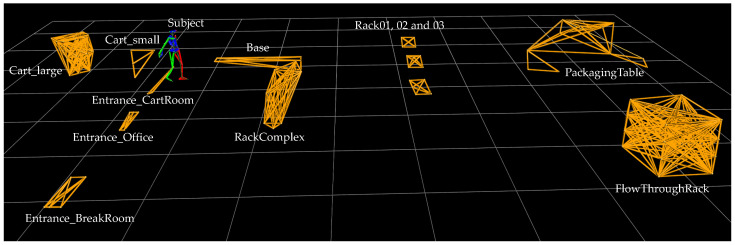
Entire laboratory set-up as an oMoCap visualisation.

In addition to the two subjects, 12 different objects were instrumented and recorded, including a base, two picking carts, entrances to rooms, various racks and a packaging table. The objects were not used equally in all three scenarios. Nevertheless, all marked objects, i.e., also unused objects, were recorded. Consequently, all files in the dataset are structured in the same way, regardless of the scenario assignment. The laboratory set-up for the three scenarios is visualised in Figure 3.

In order to capture rigid objects using the oMoCap, several prerequisites must be met. Each object must have at least three markers that form an individual pattern and are neither obscured nor change their position in relation to each other during the recording. The robustness of the estimation is increased with a larger number of visible markers. This resulted in a total number of 94 reflective markers for the 12 objects. A complete list of all objects captured by oMoCap and their scenario assignment can be seen in Table 2.

Before recording, the individual marked patterns were stored in Nexus software. By means of the stored patterns, the captured markers can be assigned to the respective objects, and thus the object can be recognised (see Figure 3). The advantage is that not only is the object recognised, but also its markers are individually named and recognised. This makes it possible to mark and recognise sub-components within an object that are too small to be equipped with sufficient markers as an independent object. For example, the large picking cart had markers that identified the handles, the individual levels in which the boxes were located and even the buttons on the put-to-light frame. The same applied to the packaging table. Instead of marking a barcode roll, a single marker was placed near the roll, which, in combination with other markers, identified the packaging table.

**Figure 4:**
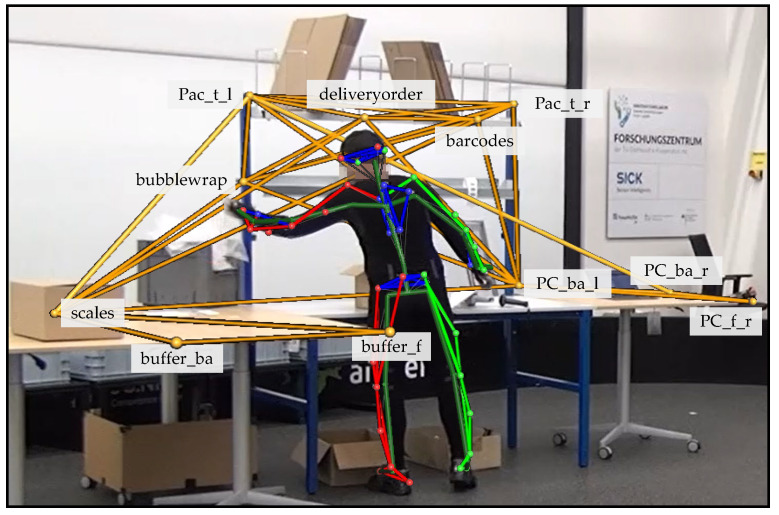
Marker designations of the packaging table.

Like the barcodes, the computer and the delivery notes, the bubble wrap was not an independent object but a sub-component of the packaging table. By combining the context information of these sub-components and the movement data of the subject, activities can be derived. If there was a hand marker near the marker *bubblewrap*, a handling activity of a small item could be assumed. Since bubble wrap is considered a small item when grabbing, but when filling the shipping box, the grabbing movement shown in Figure 4 is likely to be followed by a handling activity with *Utility* as an attribute.

### 3.4. Recordings

The recordings of both subjects took place on the same day. Neither the set-up nor the settings of the recordings were changed. Each scenario was carried out without breaks or other interruptions, with the aim of not interrupting the flow of movement of the subjects at any time. Experts in the field of logistics explained the process steps before and during each scenario. No instructions were given on how exactly to perform the movements in order to ensure the most natural body movements possible. For example, the subject decided for himself/herself whether to pick up an item with his/her left or right hand.

During the performance of picking and packaging tasks, the oMoCap system recorded the positions of markers of objects and subjects at a sampling rate of 200 fps. The processes were recorded in two-minute sequences. After all recordings were completed, time-consuming reconstruction and labelling of the markers took place. This allowed the recordings to be made immediately one after the other. Thus, there were only minimal time offsets of only a few seconds between the two-minute sequences. Both subjects were recorded 35 times for two min. This corresponds to 140 min of data.

### 3.5. Annotation Results

The annotation consisted of two steps. First, the oMoCap data were divided into one-second (200 frames) windows and automatically classified using a temporal convolutional neural network [39]. Then, two researchers used an annotation tool to manually modify the data. In total, the revision took 44.72 person-hours. This corresponds to 19.17 minutes per 1 minute recording.

All data were labelled and grouped into seven activity classes. The activity classes include locomotion, such as *Standing* ($c_{1}$), *Walking* ($c_{2}$) and *Cart* ($c_{3}$). In total, they represent about 30% of the entire dataset. Almost 70% of the activities involved *Handling* ($c_{4}$–$c_{6}$). *Handling centred* ($c_{5}$) is the most strongly represented activity. This high proportion is partly due to scenario three. Most of the movements were performed on the packaging table and thus at stomach and chest height. The *None* class ($c_{7}$) represents movements that could not be assigned to any of the first six activities and oMoCap data that incorrectly represent the subject. Table 3 gives an overview of the seven activity classes and their proportions in the dataset. On average, the annotated windows are 1.46 s long.

The activity classes are subdivided into 19 binary descriptions in more detail, also called attributes [31]. Each window is labelled with attributes describing the movement of the legs, upper body, and hands, along with information about the posture of the item:

- Legs: Gait Cycle, Step, Standing Still.
- Upper Body: Upwards, Centred, Downwards, No Intentional Motion, Torso Rotation.
- Handedness: Right Hand, Left Hand, No Hand.
- Item Pose: Bulky Unit, Handy Unit, Utility/Auxiliary, Cart, Computer, No Item.
- Other attributes: None, Error.

**Figure 5:**
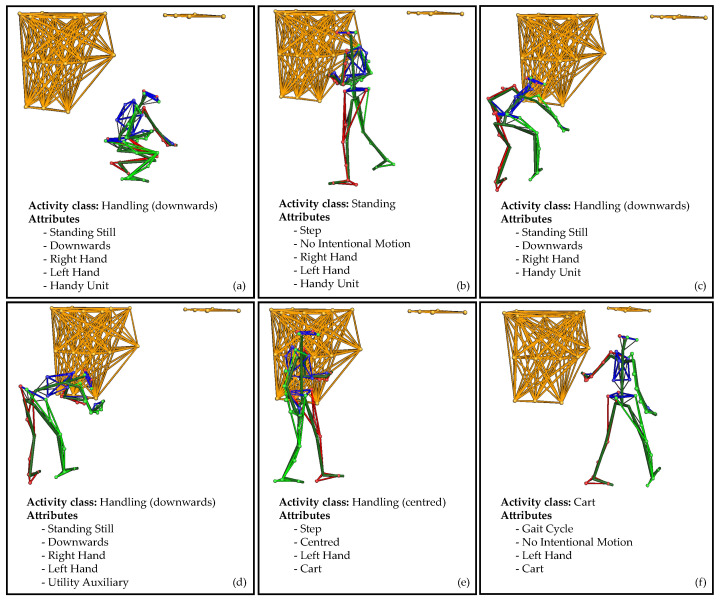
Example of an annotation sequence of six windows.

An example of an annotated sequence is visualised in Figure 5. The 20 s sequence of the second scenario consists of six windows. Each window symbolises an activity class composed of independent attribute representations. In the first window (a), the subject picks up a *small item* from the lowest level of the rack with their *right* and *left hand*. The markers (see top right) indicate the top level of the rack. Then, the subject moves with the *item* to the front of the picking cart (b). The cart is shown at the top left. Since the subject only takes two *steps*, this is not a gait cycle. The item was held by *both hands* but was not handled. This makes it the class *standing*. In the next window (c), the *item* is placed in the lowest level of the cart with the *right hand*. The release of the item is confirmed by pressing a button on the put-to-light frame of the cart (d). The button is classified as a *utility*. Both movements (c and d) are performed in a *bent posture*. The subject then grips the *cart* with the *left hand* (e). The upper body is in an straight position. In the last window, the subject *moves* with the *cart* around the rack to the next position. The cart is pulled with the *left hand without an intentional motion*, only a steady stance.

Due to the numerous possible combinations, the dataset contains a total of 122 unique attribute representations. The list of all attribute representations is given as part of the publicly available dataset. The activity classes and attributes used in this paper are explained in detail in [8] (pp. 15–19).

## 4. Activity Recognition Methodology

**Figure 6:**
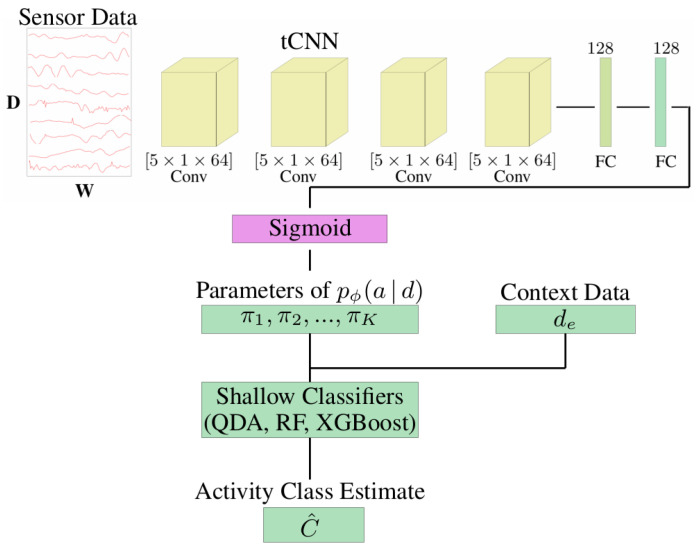
Activity recognition architecture. The pre-trained neural network predicts high-level movement descriptors (attributes). Together with the context data, they are used by a shallow classifier to predict activity classes. Figure adapted from [33].

In the following, we present the utilised HAR method, which is based on the architecture proposed by [33]. It can make use of motion data from the subject and additional context data to predict activity classes. Specifically, the architecture consists of a pre-trained deep neural network that predicts movement descriptors (attributes) from the subjects’ sensor data and a shallow classifier that predicts activity classes from these attributes and the context data (see Figure 6). The architecture is designed to be easily adaptable to different context data without having to re-train the entire model.

The deep neural network is a *temporal convolutional neural network* (CNN) [27]; i.e., its convolutional layers perform convolutions along the time axis. The CNN processes segments of 200 samples, i.e., 1 s of data due to the sampling rate of 200 Hz. The network consists of four convolutional layers, followed by two fully connected layers. The output layer has 19 units, corresponding to 19 movement attributes, and a sigmoid activation function. We did not perform any training of the network, but used the pre-trained network available at [17], which was trained on the LARa [8] dataset.

Due to the final sigmoid layer, a network output can be interpreted as the probability of an attribute being present or not present in the input segment. More formally, the network is a function ϕ:d→π, where *d* is a motion data segment and $\pi =\pi_{1},\cdots ,\pi_{19}$ are the parameters of the posterior distribution P(a|d) over attribute vectors $a=a_{1},\cdots ,a_{19}$. Specifically, the distribution over binary attribute vectors is given by a product of Bernoulli distributions:

(1) $$P_{\phi }\left(a_{1},\cdots ,a_{19}|d\right)=\prod_{i=1}^{19}\pi_{i}^{a_{i}}\;{\left(1-\pi_{i}\right)}^{1-a_{i}}$$

The overall goal of our HAR method is to estimate a posterior distribution over activity classes P(c|d) (from which estimating the maximum-a-posterior class becomes straightforward). Given a distribution P(c|a) that associates activity classes *c* and attributes, the activity class posterior P(c|d) can be computed by marginalising the attributes *a*. Further simplifications are possible when a deterministic relationship g(a)=c exists between class and attribute [33]. However, in this way, the additional data representing the context cannot be directly integrated into the activity class estimation.

Therefore, we follow the approach outlined in [33]: The network output π, along with the context data $d_{e}$, are used as input for a (shallow) classifier that predicts an activity class. We experimented with quadratic discriminant analysis (QDA), gradient boosted decision trees (XGBoost) and random forests (RF) as shallow classifiers. Alternatively, another fully connected layer can be used. The shallow classifier was trained using the pre-computed network outputs π and the context data $d_{e}$.

The underlying intuition for this architecture is the fact that processing movement data from the subject requires elaborate models to extract relevant features, but is domain-independent (using a fixed subject instrumentation). Thus, a pre-trained network can be used to save training time and data to train this part of the model. The integration of context data, on the other hand, depends on the domain, e.g., on the specific configuration of the environment, and thus the part of the model concerned with the context data is trained. Training of the final classifier can be seen as a form of *fine-tuning*, a common technique in deep learning.

The architecture has several additional advantages, compared to training an activity recognition classifier from scratch: A posterior distribution P(a|d) over attributes is available and can be used for downstream tasks. Furthermore, using such attributes as an intermediate representation has been shown to lead to higher activity recognition performance, compared to directly predicting activity classes [31].

## 5. Experimental Evaluation

### 5.1. Experimental Procedure

The goal of the experimental evaluation was to investigate the effect of the additional context data on HAR performance, in comparison to a baseline HAR method based solely on movement data. Specifically, we investigated the following three research questions:

Q1 What influence does context data have on HAR performance (in terms of F1 score), compared to a baseline model where this context data are not available?
Q2 Which context features are most relevant for HAR performance, i.e., increase F1 score the most?
Q3 How comprehensive do context data have to be? How do complex context data affect the HAR performance, compared to a model where only simple, distance-based context features are used?

To evaluate Q1, we performed ablation studies on the architecture described in Section 4: The baseline model only used the attribute estimates from the pre-trained attribute classifier [8]. We investigated three shallow classifiers for combining attribute and context data: QDA, XGBoost and RF. We used two variants of context data to answer Q3: (i) the raw, segmented data from the environmental markers, and (ii) pre-computed distances between subject markers and environmental markers. Specifically, we computed pairwise distances between 25 selected environmental markers (related to the cart, table and different racks) and the left-hand, right-hand and chest markers of the subject. The complete list of involved markers is part of the published dataset. This preprocessing was based on our intuition that activities often depend on the proximities of the subject to certain objects or locations. For example, when the subject’s hand is close to the handle of the cart, the *Cart* activity is likely.

The context data (raw marker positions and distances) were preprocessed by computing the mean of each data column for segments of length 1 s, without overlap. The attribute classifier also computes attribute estimates for 1 s segments. Thus, all inputs of the final classifier (attribute estimates and context data) were available with a sampling rate of 1 s and could be combined directly. Performance was assessed using a 10-fold cross validation, for which we made sure that all data of an experimental run were contained in the same fold. We report class-wise F1 scores and the macro F1 score.

Research question Q2 was assessed by a greedy stepwise feature selection approach: We started with the baseline model that used only the attribute estimates. At each step, the algorithm then selected the single context feature that increased macro F1 score the most, using the same 10-fold cross validation procedure as all models outlined above. This feature was added to the model, and the process was repeated until no feature increased HAR performance further.

Evaluations were performed using the R programming language [40] and the R packages xgboost [41] and randomForest [42].

### 5.2. Results

Table 4 shows the macro F1 scores of the different models. The RF outperformed QDA in all cases, and XGBoost in all cases except for the case where only distance features were available. Note that the QDA could not be applied to the raw data because of the dimensionality of the data. Thus, in the following, we focus on the RF.

The baseline RF model (using only the attribute estimates, but no context data) achieved a macro F1 score of 0.716. Adding context data improved this baseline result: attributes and distance data achieved an F1 score of 0.733, and attributes and raw context data even achieved an F1 score of 0.820. Thus, pre-computation of distance features did not seem to be beneficial in our case, and instead, working directly with the raw data provided better results. Interestingly, using just the raw context data but no attribute estimates already outperformed the baseline model. Nevertheless, the combination of both models (i.e., using attribute estimates and raw context data) improved performance compared to the former cases.

The class-wise F1 scores (Table 5) show a large variance in recognition performance for the different classes. Specifically, the F1 score for the *Standing* class was low, independently of the used data. This result could be expected because the stand class contains unspecific behaviour that does not fall in any of the other categories and is thus difficult to discriminate from the other classes. Using raw context data improved F1 scores of three classes (*Cart*, *Handling upwards*, *Walking*) with respect to the baseline model, but decreased the F1 scores of the other three classes (*Handling centered*, *Handling downwards*, *Standing*). Despite this, the overall (macro) F1 score increased significantly.

Table 6, Table 7 and Table 8 show the results of the greedy feature selection. Note that results for the base models do not agree exactly with the baseline models in Table 4 due to the random assignment of data to folds in the cross validation procedure. Consistently, only few features were selected before there was no single feature that increased F1 score further. This behaviour can be explained by the fact that the greedy feature selection approach only converges to a local minimum. Therefore, interactions of features that would increase performance further cannot be considered.

Thus, overall we could show that including few select context features already increased HAR performance, compared to the baseline model. Overall, the most relevant markers were markers related to the carts and the table, which intuitively makes sense, because knowing that the cart is moving should help identify the *Cart* activity and proximity of the subject to the table indicates handling activities. Nevertheless, the use of *all* recorded context features further improves HAR performance. Thus, the choice of utilised context features is subject to a trade-off between the cost of recording and accuracy required for downstream tasks.

## 6. Discussion and Conclusions

In this paper, we demonstrated that making use of context information can increase activity recognition performance substantially. Specifically, we recorded an oMoCap dataset that includes both the movement of subjects and the location of objects and environmental features. We analysed the dataset regarding (a) the potential of context information to increase activity recognition performance and (b) the most informative context features. We found that for our intralogistics scenarios, the location of the handle of the cart and the packaging table were most informative for activity recognition. These findings intuitive make sense, because proximity to the packaging table or cart indicate, which process step is currently performed, and thus which activities are likely to happen. Therefore, in further research, one can focus on these context information, instead of instrumenting additional objects. Since the processes in intralogistics are standardised, the results can be applied to different warehouses. However, for other tasks in the same domain, e.g., driving a forklift truck, further context data can be collected in addition to the existing ones. To account for this, our proposed architecture makes it straightforward to change the utilised context information, without re-training the entire model.

Still, further research is necessary to explore the full potential of context information for activity recognition: First, we rely on a relatively simple activity recognition architecture. Using a more elaborate model instead of the shallow classifier that is currently used, e.g., a model that allows end-to-end training of the overall model, could increase activity recognition performance further. Secondly, investigating the utility of context information for other tasks, e.g., for recognising *movement attributes* instead of activities, is an interesting direction for future work. Third, we focused on additional markers on objects and environmental features as context information. However, context information in a more general sense could contribute towards activity recognition—e.g., events occurring in the warehouse management system; prior knowledge about causal relations between activities; or additional sensors, such as inertial measurement units or cameras. Finally, we focused on using context information to improve activity recognition, but one could also be interested in inferring the context (e.g., the currently used object) from the subject’s movement or recognised activities. The long-term goal of this research direction is to jointly estimate the model of motion attributes, activities and context from multiple sensor modalities (e.g., inertial measurement units, Bluetooth low energy devices and radio-frequency identification for industrial application) and prior domain knowledge.

## Figures and Tables

**Table 1:** Subjects—specifications and scenario assignment.

ID	Sex	Age	Weight	Height	Handedness	No. of Two-Minute Recordings		
	[F/M]	[years]	[kg]	[cm]	[L/R]	L01	L02	L03
S17	M	30	85	176	R	5	15	15
S18	F	26	62	177	R	5	15	15

**Table 2:** Objects—specifications and scenario assignment.

Name in	No. of	Logistics Scenario		
Dataset Files	Marker	L01	L02	L03
Base	5	x		
Cart_large	15		x	
Cart_small	4	x		
Entrance_BreakRoom	5	x	x	x
Entrance_CartRoom	4	x	x	
Entrance_Office	5		x	x
FlowThroughRack	19		x	x
PackagingTable	11			x
Rack01	5	x	x	
Rack02	5	x	x	
Rack03	5	x	x	
RackComplex	11	x	x	

**Table 3:** Annotation results divided by activity classes.

Activity Class		Frames		Windows			
		No.	%	No.	%	Min. Length [Frames]	Max. Length [Frames]
$c_{1}$	Standing	112,228	6.68	432	7.51	32	2348
$c_{2}$	Walking	181,596	10.81	326	5.67	61	8800
$c_{3}$	Cart	207,774	12.37	315	5.48	60	4400
$c_{4}$	Handling (upwards)	137,911	8.21	589	10.24	26	1400
$c_{5}$	Handling (centred)	954,959	56.84	3732	64.90	26	4607
$c_{6}$	Handling (downwards)	72,368	4.31	339	5.90	29	1384
$c_{7}$	None	13,164	0.78	17	0.30	200	2600
		1,680,000	100	5750	100		

**Table 4:** Macro F1 scores of the different classifiers and feature subsets.

Classifier	Att. (Base Model)	Dists	Att. + Dists	Raw	Att. + Raw
QDA	0.670	0.515	0.619	–	–
XGBoost	0.717	0.730	0.643	0.691	0.727
RF	0.716	0.646	0.733	0.745	0.820

**Table 5:** Class-wise F1 scores of RF model.

	Standing	Walking	Cart	Handling	Handling	Handling
				Upwards	Centred	Downwards
Att. (Base model)	0.210	0.743	0.809	0.880	0.761	0.815
Dists	0.063	0.716	0.819	0.858	0.508	0.593
Att. + Dists	0.156	0.842	0.880	0.886	0.743	0.822
Raw	0.214	0.823	0.803	0.808	0.687	0.804
Att. + Raw	0.150	0.833	0.880	0.825	0.753	0.826

**Table 6:** Greedy feature selection results (QDA, distance features).

Marker 1	Marker 2	F1 Score
Attributes (Base model)		0.680
+ Cart_large:C_large_PtL_3_l	Subject:RFIN	0.698
+ PackagingTable:barcodes	Subject:RFIN	0.705
+ Cart_small:C_small_handle	Subject:RFIN	0.711
+ Cart_small:C_small_ba_t	Subject:RFIN	0.718
+ Cart_small:C_small_ba_t	Subject:LFIN	0.719
+ PackagingTable:bubblewrap	Subject:LFIN	0.719

**Table 7:** Greedy feature selection results (RF, distance features).

Marker 1	Marker 2	F1 Score
Attributes (Base model)		0.717
+ PackagingTable:barcodes	Subject:LFIN	0.740

**Table 8:** Greedy feature selection results (RF, raw features).

Marker	Axis	F1 Score
Attributes (Base model)		0.717
+ PackagingTable:Pac_t_r	z	0.748
+ Cart_small:C_small_handle	y	0.761

## Data Availability

The dataset used in this work is freely available: [16].
